# Supplementary material for: Cognitive functioning and lifetime major depressive disorder in UK Biobank
Source: Eur Psychiatry. 2020 Feb 21;63(1):e28. doi: 10.1192/j.eurpsy.2020.24 (PMC7315876; doi:10.1192/j.eurpsy.2020.24)
Supplement: Supplementary file 1 [file S0924933820000243sup001.docx]

**SUPPLEMENTARY MATERIALS**

**Cognitive functioning and lifetime Major Depressive Disorder in UK Biobank**

Supplementary Methods

1. Participant exclusions (Figure S1)
2. Putative current definition
3. Classes of psychotropic medication (Figure S2)
4. Education, smoking and alcohol consumption variables

Supplementary Results

1. *G*-factor component loadings and correlations (Table S1, Figure S3)
2. Cognitive sample sizes and descriptive statistics (Table S2)
3. Associations between cognitive performance and covariates (Table S3)
4. Exploration of age by MDD interactions on cognitive functioning (Table S4)
5. Exploration of parental history effects on cognitive functioning (Table S5)
6. Cognitive profiles associated with clinical characteristics (Table S6)

Supplemental References

1. **Participant exclusions**

*Exclusion of participants with neurological conditions*
 For the purpose of the present study, participants were excluded from all analyses if they reported having received a diagnosis of dementia and/or cognitive impairment, head or neurological injury or trauma, Parkinson’s disease, Multiple Sclerosis or other demyelinating disease, or any other chronic/degenerative neurological problem.

*Exclusion of incomplete observations* The measure of general cognitive performance (*g*-factor) was derived including only complete cognitive testing observations.

*Exclusion of participants with specific psychiatric disorders* Participants were excluded from case-control analyses if they had received a diagnosis of bipolar disorder, schizophrenia, Parkinson’s disease, autism or intellectual disability, or if they were using antipsychotic medication, as reported at any assessment centre visit or on the MHQ.

*Missing age data* In UK Biobank, the age of the participant at time of the assessment is a derived measure, derived from baseline data on birth month and year and the date of the assessment centre visit. For some participants, the date of the third assessment centre visit was missing, so that the age of the participant was unknown.

*Unclassified lifetime MDD status*
 Participants were unclassified if one of the following three situations applied (1):

1. They did not meet lifetime MDD criteria, but did self-report a diagnosis of MDD
2. They met part of the criteria for lifetime MDD, so that classification would be ambiguous; The participant had either experienced a period of depressed mood and/or loss of interest, during which they felt this way (at least) on most days of the week for (at least) most of the day (criterium ii in main text), which was causing impairments in psychosocial functioning (criterium iii in main text), but did not have a total symptom score of at least five (criterium i in main text), or did have a symptom score of at least five, but did not meet the criteria of frequency of negative feelings (ii) and/or psychosocial impairment (iii).
3. They reported one or more periods of depressed mood and/or loss of interest, but had missing data with regard to any of the follow-up question that are used to assess the three criteria for lifetime MDD classification.

*Control group exclusion criteria* Additional exclusion criteria for the control group were a reported mood or anxiety disorder diagnosis, or reported use of psychotropic medication, at any of the UK Biobank assessments.

**
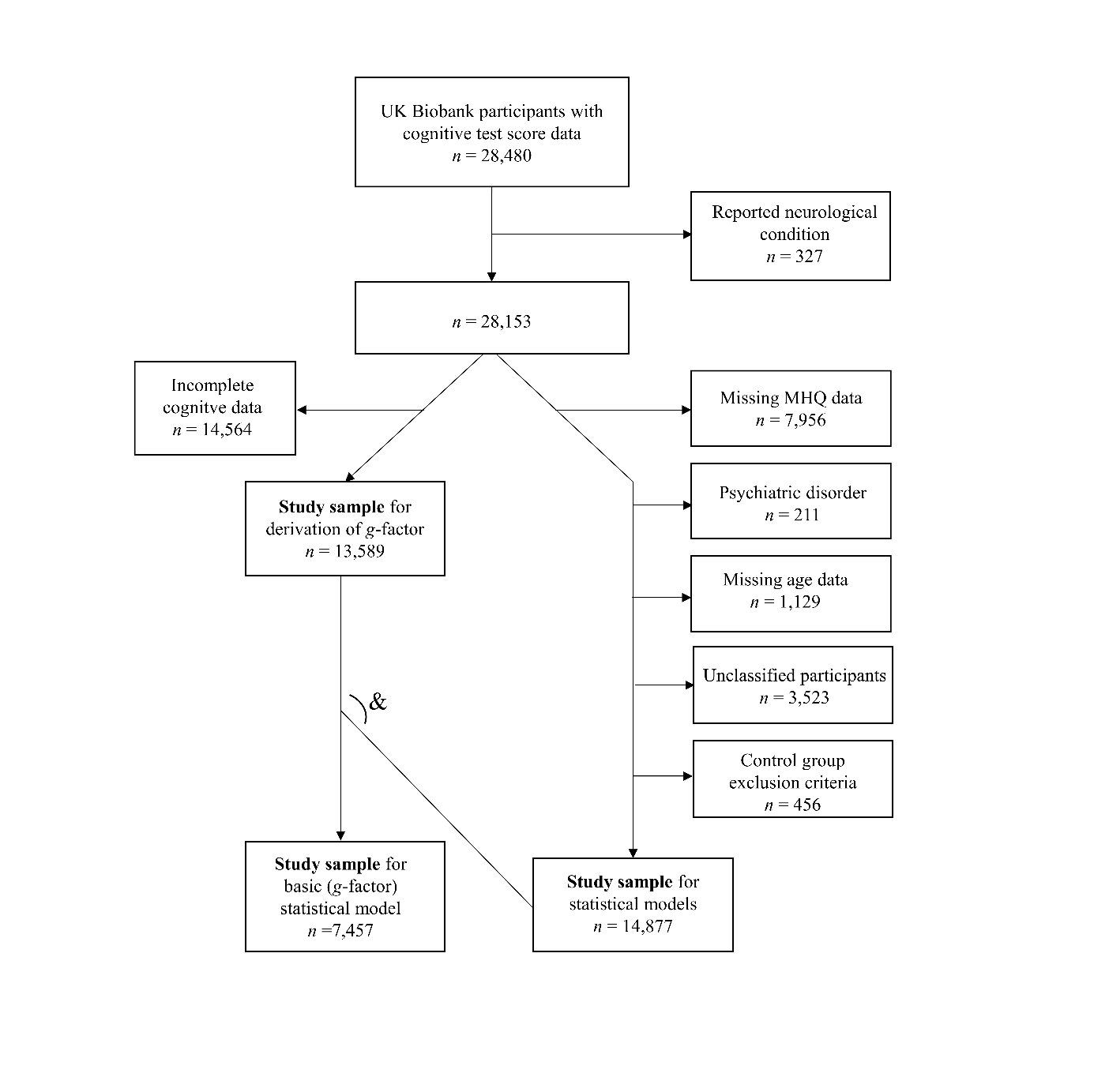
*Figure S1.* Flow-chart of participant exclusions**.
This chart displays the procedure of participant exclusion that was used to compose the study samples of the present study. The study sample for the basic (*g*-factor) statistical model consisted of the participants who were included in the total study sample for statistical models and had complete cognitive test data. Hence, this study sample was the smallest sample within our study.

1. **Putative current definition**

Since UK Biobank touchscreen questionnaires lacked a validated and complete assessment of current MDD symptomatology, the ‘putative current’ definition was locally derived in order to examine associations with possible MDD symptomatology at the time of the cognitive assessment (i.e., third UK Biobank assessment). The ‘putative current’ definition provided a binary classification of participants who met lifetime MDD criteria, with no unclassified cases. Overall, the criteria for the definition were chosen intuitively, and so that appropriate group numbers were achieved (i.e., within the overall UK Biobank sample, for use in future analyses). The criteria for ‘putative current’, of which only one needed to apply to the case participant, were the following:

1. Depressed mood over the past two weeks, “More than half of the days” or “Nearly every day”
2. Anhedonia over the past two weeks, “More than half of the days” or “Nearly every day”
3. General happiness, “Very unhappy” or “Extremely unhappy”
4. Experiencing other (or less severe) mood, sleep, psychomotor and interpersonal symptoms.

Mood, sleep, psychomotor and interpersonal symptoms (from criterium iv) formed separate symptom criteria groups, containing the criteria described below. In order to meet criterium (iv), the participant needed to meet at least one criterium within at least three out of the four symptom criteria groups.

Mood symptoms

- Often feeling miserable, “Yes”
- Often feeling fed up, “Yes”
- Depressed mood over the past two weeks, “Several days”
- Anhedonia over the past two weeks, “Several days”
- General happiness, “Moderately unhappy”

Sleep problems

- Experience of getting up in the morning, “Not very easy” or “Not at all easy”
- Troubled sleeping, “Usually”

Psychomotor symptoms

- Feelings of restless over the past two weeks, “More than half of the days” or “Nearly every day”
- Feelings of tiredness over the past two weeks, “More than half of the days” or “Nearly every day”

Interpersonal problems:

- Irritable person, “Yes”
- Sensitive person (i.e., feelings are easily hurt), “Yes”
- Often feeling lonely, “Yes”
- Often troubled by feelings of guilt, “Yes”

1. **Classes of psychotropic medication**

**
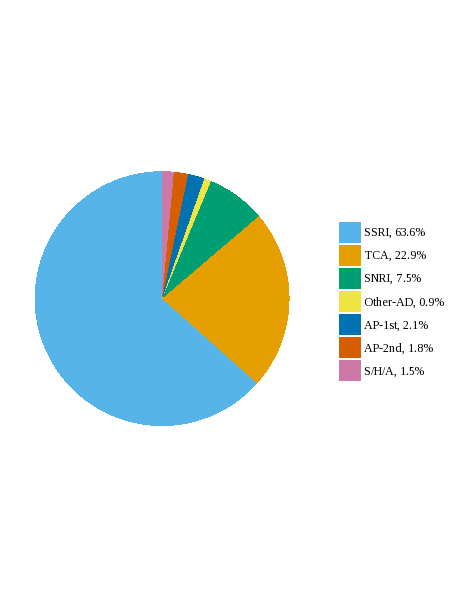
**

AD, antidepressant; AP-1^st^, first generation antipsychotic; AP-2^nd^, second generation antipsychotic; TCA, tricyclic/tetracyclic antidepressant; S/H/A, sedative/hypnotic or anticonvulsant medication; SNRI, Selective Norepinephrine Reuptake Inhibitor; SSRI, Selective Serotonin Reuptake Inhibitor.

***Figure S2.* Classes of psychotropic medication in case sample.**Pie chart representing the psychotropic medication (*n* = 332 data entries) reported by case participants, as categorised in classes. In total, case participants (*n* = 313) reported the use of 24 different medication types of the classes antidepressant (SSRI, SNRI, TCA or other), antipsychotic (1^st^ or 2^nd^ generation), and sedative/hypnotic/anticonvulsant medication. Most case participants reported current use of one type of psychotropic medication (*n* = 294), but combinations of two different antidepressants (*n* = 13), of an antidepressant with an antipsychotic (*n* = 4) and of an antidepressant with an hypnotic/sedative/anticonvulsant (*n* = 2) were also reported.

1. **Education, smoking and alcohol variables**

*Education* Education was assessed as part of the touchscreen questionnaire with a multiple choice question that allowed participants to report all qualifications. Participants were classified in one of five categories representing the (highest) type of education that they had received: University education (“College or University degree”, college education (“NVQ or HND or HNC or equivalent” or “other professional qualifications, e.g. nursing or teaching”), continued education (“A levels/AS levels or equivalent”), compulsory education (“CSEs or equivalent” or “O levels/GCSEs or equivalent”) or incomplete education (“None of the above”).

*Alcohol consumption*
 In the current study, we calculated standard alcohol units per week from reported average weekly and monthly intake of various alcoholic drinks (2). As part of the touchscreen questionnaire, participants were asked to provide how many glasses of each alcoholic drink they consumed either per week or per month, depending on the reported frequency of alcohol intake. These questions were supported by an image of each alcoholic drink that indicated the magnitude of the portion (e.g., a glass of wine or pint of beer). The alcoholic drinks listed below were converted to standard alcohol units using appropriate scaling factors, in order to calculate the total intake of standard alcohol units (on average) per week:

1. Red wine intake (scaling factor 1.67)
2. White wine and champagne intake (scaling factor 1.67)
3. Beer and cider intake (scaling factor 2.3)
4. Spirit and liqueur intake (scaling factor 1)
5. Fortified wine intake (scaling factor 2.25)
6. Intake of other alcoholic drinks (scaling factor 1.1)

*Lifetime smoking* Smoking was represented by a categorical variable, derived from history of regular smoking and the lifetime amount of smoked cigarettes. The variable pack years had been previously derived for participants who reported to be a current cigarette smoker (either manufactured or hand rolled) and to smoke on most days, or to have previously smoked these on most days (Field name: 20161; Wain *et al.* 2015). Pack years of smoking represents how many packs of cigarettes a (previous) regular smoker has smoked in his life, and is calculated as the packs of cigarettes smoked per day (number of cigarettes divided by twenty) multiplied by the number of years the person has smoked. For each of the analyses considering lifestyle, participants with a calculated value for pack years were classified in one of four smoking categories based on quantiles, while participants who never smoked cigarettes on a regular basis constituted a separate category.

1. ***G*-factor component loadings and correlations**

|  | **Loading** |
| --- | --- |
| Trail Making Test B (TMT-B) | 0.47 |
| Verbal Numeric Reasoning (VNR) | 0.42 |
| Matrix pattern completion (Matrix) | 0.42 |
| Digit Symbol Substitution Task (DSST) | 0.40 |
| Tower rearranging (Tower) | 0.39 |
| Numeric Memory (NM) | 0.34 |

*Table S1*. Principal component loadings of cognitive tests on *g­*-factor

**
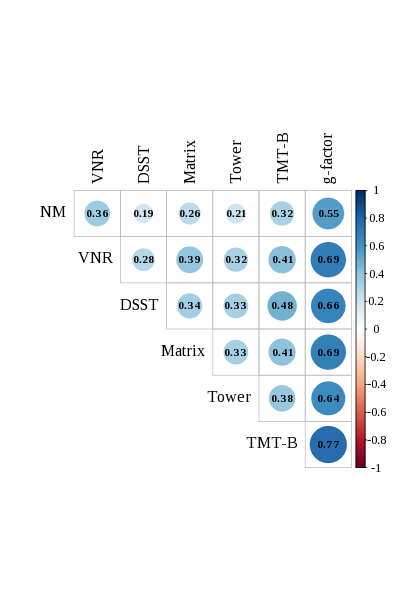
**

DSST, Digit Symbol Substitution Task; *g*-factor, derived measure of general cognitive performance; Matrix, Matrix pattern completion; NM, Numeric Memory; TMT-B, Trail Making Test, alphanumeric trail; Tower, Tower rearranging; VNR, Verbal Numeric Reasoning.

***Figure S3.* Correlation matrix of cognitive tests scores and g-factor.**
This figure graphically displays Pearson correlation coefficients between cognitive tests scores and the *g*-factor. All correlations were highly significant (*P* < 2.2×10^-16^) and in the expected direction. Of note, the variable TMT-B was reversed so that higher scores represented better performance. For more information on the cognitive tests and test score variables, see Table 1 in main text.

1. **Cognitive test sample sizes and descriptive statistics**

*Table S2.* Cognitive test sample sizes and descriptive statistics.

| **Test** | **Test score variable** | **Sample sizes (*n*)** | | **Descriptive Statistics** | |
| --- | --- | --- | --- | --- | --- |
|  |  | Control group | Case group | Control group | Case group |
| DSST^a^ | Total correct score | 5,468 | 2,318 | 19.3 (5.2) | 19.3 (5.1) |
| TMT-B^b^ | Time to completion (s) | 5,348 | 2,285 | 49.8 (22.4) | 49.6 (21.2) |
| NM^a^ | Maximum digits | 5,789 | 2,469 | 6.9 (1.3) | 6.8 (1.2) |
| Matrix^a^ | Total correct score | 5,444 | 2,315 | 8.1 (2.1) | 8.1 (2.0) |
| VNR^a^ | Total correct score | 10,360 | 4,476 | 6.9 (2.1) | 6.8 (2.0) |
| Tower^a^ | Total correct score | 5,414 | 2,296 | 10.1 (3.2) | 10.0 (3.2) |

*Note: This table displays raw cognitive scores. TMT-B scores were transformed and reversed before statistical analysis.*
DSST, Digit Symbol Substitution Task; Matrix, Matrix pattern completion; NM, Numeric Memory; TMT-B, Trail Making Test, alphanumeric trail; Tower, Tower rearranging; VNR, Verbal Numeric Reasoning.
^a^ Descriptive statistics are mean (SD)
^b^ Descriptive statistics are median (IQR)

1. **Associations between cognitive performance and covariates**

Associations between cognitive performance and covariates can be derived from the linear models by considering the corresponding test statistics (Table S3). Briefly, increased age was associated with lower general cognitive performance (*g*-factor) and lower performance on all cognitive tests. Males showed a higher *g*-factor than females and performed better on most cognitive tests (NM, Matrix, VNR, Tower). Increased education was consistently associated with higher cognition, showing increasingly better performance from incomplete education to compulsory, college, continued and university education. BMI showed a consistent negative association with general cognitive performance and individual test scores (except Tower). On the other hand, alcohol units per week was positively associated with general cognitive performance and performance on NM, VNR and Tower. With regard to smoking, associations suggested lower processing speed for individuals from the heaviest smoking category (see findings of DSST and TMT-B), while individuals from the lightest smoking category slightly outperformed the reference group of individuals who never smoked regularly.

*Table S3.* Linear model *β*-coefficients (SE) of covariates

|  | **Model 1** | | | | | | |
| --- | --- | --- | --- | --- | --- | --- | --- |
|  | ***g-factor*** | ***DSST*** | ***TMT-B*** | ***NM*** | ***Matrix*** | ***VNR*** | ***Tower*** |
| Age (years) | -0.05 (0.00)*** | -0.06 (0.00)*** | -0.05 (0.00)*** | -0.02 (0.00)*** | -0.03 (0.00)*** | -0.01 (0.00)*** | -0.03 (0.00)*** |
| Sex, Male | 0.15 (0.02)*** | -0.03 (0.02) | -0.02 (0.02) | 0.22 (0.02)*** | 0.18 (0.02)*** | 0.15 (0.02)*** | 0.17 (0.02)*** |
|  | **Model 2** | | | | | | |
|  | ***g-factor*** | ***DSST*** | ***TMT-B*** | ***NM*** | ***Matrix*** | ***VNR*** | ***Tower*** |
| Age (years) | -0.05 (0.00)*** | -0.06 (0.00)*** | -0.05 (0.00)*** | -0.02 (0.00)*** | -0.03 (0.00)*** | -0.01 (0.00)*** | -0.03 (0.00)*** |
| Sex, Male | 0.14 (0.02)*** | -0.03 (0.02) | *-*0.03 (0.02) | 0.21 (0.02)*** | 0.17 (0.02)*** | 0.13 (0.02)*** | 0.17 (0.02)*** |
| Education^a^ |  |  |  |  |  |  |  |
| *Incomplete* | -0.67 (0.06)*** | -0.34 (0.05)*** | -0.48 (0.06)*** | -0.47 (0.06)*** | -0.42 (0.06)*** | -0.55 (0.04)*** | -0.38 (0.06)*** |
| *Continued* | 0.37 (0.05)*** | 0.21 (0.05)*** | 0.25 (0.05)*** | 0.18 (0.05)*** | 0.30 (0.05)*** | 0.38 (0.04)*** | 0.12 (0.06)* |
| *College* | 0.13 (0.04)*** | 0.05 (0.04) | 0.06 (0.04) | -0.03 (0.04) | 0.19 (0.04)*** | 0.16 (0.03)*** | 0.06 (0.04) |
| *University* | 0.51 (0.03)*** | 0.20 (0.03)*** | 0.28 (0.03)*** | 0.18 (0.03)*** | 0.55 (0.03)*** | 0.60 (0.02)*** | 0.19 (0.04)*** |
|  | **Model 3** | | | | | | |
|  | ***g-factor*** | ***DSST*** | ***TMT-B*** | ***NM*** | ***Matrix*** | ***VNR*** | ***Tower*** |
| Age (years) | -0.05 (0.00)*** | -0.06 (0.00)*** | -0.05 (0.00)*** | -0.02 (0.00)*** | -0.03 (0.00)*** | -0.01 (0.00)*** | -0.03 (0.00)*** |
| Sex, Male | 0.15 (0.02)*** | -0.02 (0.02) | -0.02 (0.02) | 0.23 (0.03)*** | 0.22 (0.03)*** | 0.13 (0.02)*** | 0.15 (0.03)*** |
| Alcohol units^b^ | 0.02 (0.01) | 0.02 (0.01) | 0.01 (0.01) | 0.03 (0.01)* | -0.01 (0.01) | 0.04 (0.01)*** | 0.03 (0.01)* |
| BMI^b^ | -0.43 (0.07)*** | -0.41 (0.07)*** | -0.20(0.07)** | -0.39 (0.07)*** | -0.40 (0.07)*** | -0.31 (0.06)*** | 0.01 (0.08) |
| Smoking^c^ |  |  |  |  |  |  |  |
| *Pack years 1* | 0.08 (0.05) | 0.00 (0.05) | 0.02 (0.05) | 0.02 (0.05) | 0.06 (0.05) | 0.02 (0.04) | 0.03 (0.05) |
| *Pack years 2* | -0.12 (0.05)* | -0.07 (0.05) | -0.07 (0.05) | -0.02 (0.05) | -0.15 (0.05)** | -0.02 (0.04) | -0.03 (0.05) |
| *Pack years 3* | -0.02 (0.05) | -0.05 (0.05) | -0.02 (0.05) | 0.07 (0.05) | -0.08 (0.05) | -0.12 (0.04)** | 0.01 (0.05) |
| *Pack years 4* | -0.13 (0.05)* | -0.21 (0.05)*** | -0.10 (0.05)* | 0.00 (0.05) | -0.17 (0.05)*** | -0.05 (0.04)* | 0.08 (0.03) |
|  | **Model 4** | | | | | | |
|  | ***g-factor*** | ***DSST*** | ***TMT-B*** | ***NM*** | ***Matrix*** | ***VNR*** | ***Tower*** |
| Age (years) | -0.05 (0.00)*** | -0.06 (0.00)*** | -0.05 (0.00)*** | -0.02 (0.00)*** | -0.03 (0.00)*** | -0.01 (0.00)*** | -0.03 (0.00)*** |
| Sex, Male | 0.14 (0.02)*** | -0.03 (0.02) | -0.03 (0.02) | 0.22 (0.02) | 0.20 (0.02)*** | 0.11 (0.02)*** | 0.14 (0.03)*** |
| Education^a^ |  |  |  |  |  |  |  |
| *Incomplete* | -0.65 (0.06)*** | -0.30 (0.06)*** | -0.47 (0.06)*** | -0.48 (0.06)*** | -0.41 (0.06)*** | -0.51 (0.04)*** | -0.34 (0.07)*** |
| *Continued* | 0.37 (0.06)*** | 0.18 (0.05)** | 0.27 (0.06)*** | 0.20 (0.06)*** | 0.28 (0.06)*** | 0.40 (0.04)*** | 0.12 (0.06) |
| *College* | 0.14 (0.04)*** | 0.05 (0.04) | 0.08 (0.04)* | -0.02 (0.04) | 0.21 (0.04)*** | 0.17 (0.03)*** | 0.07 (0.04) |
| *University* | 0.51 (0.04)*** | 0.17 (0.04)*** | 0.30 (0.04)*** | 0.19 (0.04)*** | 0.54 (0.04)*** | 0.61 (0.03)*** | 0.20 (0.04)*** |
| Alcohol units^b^ | 0.01 (0.01) | 0.01 (0.01) | 0.00 (0.01) | 0.02 (0.01)* | -0.02 (0.01) | 0.03 (0.01)*** | 0.02 (0.01)* |
| BMI^b^ | -0.25 (0.07)*** | -0.35 (0.07)*** | -0.08 (0.07) | -0.28 (0.07)*** | -0.23 (0.07)** | -0.12 (0.05)* | 0.08 (0.08) |
| Smoking^c^ |  |  |  |  |  |  |  |
| *Pack years 1* | 0.10 (0.05)* | 0.00 (0.05) | 0.03 (0.05) | 0.17 (0.05)*** | 0.08 (0.05) | 0.04 (0.03) | 0.04 (0.05) |
| *Pack years 2* | -0.07 (0.05) | -0.06 (0.05) | -0.03 (0.05) | 0.00 (0.05) | -0.11 (0.05)* | 0.07 (0.03)* | -0.01 (0.05) |
| *Pack years 3* | 0.05 (0.05) | -0.03 (0.05) | 0.02 (0.05) | 0.10 (0.05)* | -0.01 (0.05) | 0.07 (0.03) | 0.03 (0.05) |
| *Pack years 4* | 0.00 (0.05) | -0.16 (0.05)*** | -0.09 (0.05)*** | 0.06 (0.05) | -0.05 (0.05) | 0.04 (0.04) | 0.13 (0.05)* |

* *P* < .05, ** *P* < .01, *** *P* < .001
^a^ Compulsory education as reference category.
^b^ Variable was transformed, see main text.
^c^ Never regular smokers as reference category; Pack categories represent an increasing number of lifetime smoked cigarettes.
BMI, Body Mass Index; DSST, Digit Symbol Substitution Task; *g*-factor, derived measure of general cognitive performance; Matrix, Matrix pattern completion; NM, Numeric Memory; TMT-B, Trail Making Test, alphanumeric trail; Tower, Tower rearranging; VNR, Verbal Numeric Reasoning.

1. **Exploration of age by MDD interactions on cognitive functioning**

|  | **Age** | **MDD** | **Age*MDD** |
| --- | --- | --- | --- |
| ***g*-factor** | -0.38 (-0.41, -0.36)*** | -0.10 (-0.15, -0.05)*** | 0.03 (-0.01, 0.08) |
| **DSST** | -0.45 (-0.48, -0.43)*** | -0.13 (-0.18, -0.09)*** | 0.03 (-0.02, 0.07) |
| **TMT-B** | -0.40 (-0.42, -0.37)*** | -0.08 (-0.13, -0.04)*** | 0.01 (-0.03, 0.06) |
| **NM** | -0.14 (-0.16, -0.11)*** | -0.03 (-0.08, 0.02) | 0.00 (-0.04, 0.05) |
| **Matrix** | -0.24 (-0.27, -0.22)*** | -0.03 (-0.07, 0.02) | 0.01 (-0.04, 0.05) |
| **VNR** | -0.09 (-0.11, -0.07)*** | -0.06 (-0.09, -0.02)** | 0.02 (-0.01, 0.06) |
| **Tower** | -0.23 (-0.26, -0.20)*** | -0.06 (-0.11, -0.01)* | 0.04 (-0.01, 0.09) |

*Table S4.* Explorative analyses of age by MDD interactions on cognitive functioning

*Note: The table displays β-coefficient (95% confidence intervals). These explorative models included age scaled to Z-score (M = 0, SD = 1). Sex was included as covariate.** *P* < .05, ** *P* < .01, *** *P* < .001
DSST, Digit Symbol Substitution Task; *g*-factor, derived measure of general cognitive performance; Matrix, Matrix pattern completion; NM, Numeric Memory; TMT-B, Trail Making Test, alphanumeric trail; Tower, Tower rearranging; VNR, Verbal Numeric Reasoning.

1. **Exploration of parental history effects on cognitive functioning**

*Table S5.* Explorative analyses of parental history effects on cognitive functioning

|  | **Control** | **Case** |
| --- | --- | --- |
| ***g*-factor** | 0.00 (-0.09, 0.10) | -0.01 (-0.11, 0.10) |
| **DSST** | -0.05 (-0.14, 0.05) | 0.01 (-0.09, 0.12) |
| **TMT-B** | -0.02 (-0.12, 0.08) | -0.02 (-0.13, 0.08) |
| **NM** | -0.01 (-0.11, 0.10) | 0.00 (-0.11, 0.11) |
| **Matrix** | 0.02 (-0.09, 0.12) | -0.01 (-0.12, 0.11) |
| **VNR** | 0.09 (-0.01, 0.20) | 0.06 (-0.05, 0.18) |
| **Tower** | -0.02 (-0.13, 0.08) | -0.08 (-0.19, 0.04) |

*Note: The table displays β-coefficients (95% confidence intervals) of the associations between parental history of severe depression and cognitive functioning, investigated separately within the samples of control and case participants with linear models that included age and sex as covariates.** *P* < .05, ** *P* < .01, *** *P* < .001
DSST, Digit Symbol Substitution Task; *g*-factor, derived measure of general cognitive performance; Matrix, Matrix pattern completion; NM, Numeric Memory; TMT-B, Trail Making Test, alphanumeric trail; Tower, Tower rearranging; VNR, Verbal Numeric Reasoning.

1. **Cognitive profiles associated with clinical characteristics**

*Table S6.* Cognitive profiles associated with clinical characteristics

|  | **Recurrent** | **Putative current symptoms** | **Psychosocial impairment** | **Psychotropic medication** |
| --- | --- | --- | --- | --- |
| **DSST** |  |  |  |  |
| *β-*coefficient | -0.04 | -0.07 | -0.01 | -0.10 |
| (95% CI) | (-0.12,0.04) | (-0.17,0.02) | (-0.12,0.09) | (-0.21,0.01) |
| **TMT-B** |  |  |  |  |
| *β-*coefficient | -0.02 | -0.05 | -0.12* | -0.13* |
| (95% CI) | (-0.10,0.06) | (-0.14,0.05) | (-0.23,-0.02) | (-0.24,-0.01) |
| **NM** |  |  |  |  |
| *β-*coefficient | 0.02 | -0.05 | -0.06 | -0.07 |
| (95% CI) | (-0.06,0.11) | (-0.15,0.04) | (-0.17,0.05) | (-0.18,0.05) |
| **Matrix** |  |  |  |  |
| *β-*coefficient | 0.04 | -0.07 | -0.09 | -0.09 |
| (95% CI) | (-0.05,0.12) | (-0.17,0.03) | (-0.20,0.02) | (-0.21,0.03) |
| **VNR** |  |  |  |  |
| *β*-coefficient | 0.02 | -0.06 | **-0.18***** | -0.05 |
| (95% CI) | (-0.04,0.09) | (-0.13,0.00) | (-0.26,-0.10) | (-0.13,0.03) |
| **Tower** |  |  |  |  |
| *β-*coefficient | -0.01 | 0.01 | -0.05 | 0.02 |
| (95% CI) | (-0.09,0.08) | (-0.09,0.11) | (-0.17,0.06) | (-0.11,0.14) |

*Note: The model included only those individuals with a classification of lifetime MDD, so that the results reflect cognitive performance of case individuals with the clinical characteristic versus case individuals without the characteristic.** *P* < .05, ** *P* < .01, *** *P* < .001, **bold** *P_FDR_* < .05
CI, Confidence Interval; DSST, Digit Symbol Substitution Task; Matrix, Matrix pattern completion; NM, Numeric Memory; TMT-B, Trail Making Test, alphanumeric trail; Tower, Tower rearranging; VNR, Verbal Numeric Reasoning.

**Supplemental References**

1. Davis KAS, Coleman JR, Adams M, Allen N, Breen G, Cullen B, et al. Mental Health in UK Biobank Revised – development, implementation and results from an online questionnaire completed by 157,366 participants. medRxiv [preprint]. 2019;1–20.

2. Clarke T-K, Adams MJ, Davies G, Howard DM, Hall LS, Padmanabhan S, et al. Genome-wide association study of alcohol consumption and genetic overlap with other health-related traits in UK Biobank (N = 112 117). Mol Psychiatry. 2017;22:1376–84.

3. Wain L V, Shrine N, Miller S, Jackson VE, Ntalla I, Artigas MS, et al. Novel insights into the genetics of smoking behaviour, lung function, and chronic obstructive pulmonary disease (UK BiLEVE): a genetic association study in UK Biobank. Lancet Respir Med. 2015;3:769–81.
